# Retraction: Aesthetic preference for artificially selected color variant affects mate choice copying behavior in female *Poecilia latipinna*

**DOI:** 10.1371/journal.pone.0361670

**Published:** 2026-10-29

**Authors:** The PLOS One Editors

Following the publication of this article, the corresponding author made PLOS aware that the Ethics statement in the Methods section of [1] is incorrect and that the work reported in [1] was not supported by a valid protocol approval.

A representative of Merrimack College stated that a valid Institutional Animal Care and Use Committee (IACUC) approved protocol covering the experiments in [1] does not exist, that approval from an external IACUC was not sought, and therefore that the Ethics statement in the Methods section of [1] is falsified.

In light of the above concerns about adherence to PLOS’ policy on Animal Research, the *PLOS One* Editors retract this article.

The author did not respond to the final editorial decision.
